# Hybrid graphene/cadmium-free ZnSe/ZnS quantum dots phototransistors for UV detection

**DOI:** 10.1038/s41598-018-23507-y

**Published:** 2018-03-23

**Authors:** Yi-Lin Sun, Dan Xie, Meng-Xing Sun, Chang-Jiu Teng, Liu Qian, Ruo-Song Chen, Lan Xiang, Tian-Ling Ren

**Affiliations:** 1 Institute of Microelectronics, Tsinghua National Laboratory for Information Science and Technology (TNList), Tsinghua University, Beijing, 100084 People’s Republic of China; 2 Department of Chemistry, Tsinghua University, Beijing, 100084 People’s Republic of China; 3 Departmentof Chemical Engineering, Tsinghua University, Beijing, 100084 People’s Republic of China

## Abstract

Graphene-based optoelectronic devices have attracted much attention due to their broadband photon responsivity and fast response time. However, the performance of such graphene-based photodetectors is greatly limited by weak light absorption and low responsivity induced by the gapless nature of graphene. Here, we achieved a high responsivity above 10^3^ AW^−1^ for Ultraviolet (UV) light in a hybrid structure based phototransistor, which consists of CVD-grown monolayer graphene and ZnSe/ZnS core/shell quantum dots. The photodetectors exhibit a selective photo responsivity for the UV light with the wavelength of 405 nm, confirming the main light absorption from QDs. The photo-generated charges have been found to transfer from QDs to graphene channel, leading to a gate-tunable photo responsivity with the maximum value obtained at *V*_*G*_ about 15V. A recirculate 100 times behavior with a good stability of 21 days is demonstrated for our devices and another flexible graphene/QDs based photoconductors have been found to be functional after 1000 bending cycles. Such UV photodetectors based on graphene decorated with cadmium-free ZnSe/ZnS quantum dots offer a new way to build environmental friendly optoelectronics.

## Introduction

Graphene is a promosing 2D material for optoelectronics^1–3^ and photodetection applications^4–6^ due to its broad absorption bandwidth, excellent carrier transport properties and good flexibility for stretchable and wearable electronics^7,8^. However, a low absorption coefficient, which causes a barrier from photon to generated charge carriers, has limited the responsivity of graphene-based photodetectors to ~10^−2^ AW^−1^ ^9^. So far, great efforts on the achievement of high-performance graphene-based photodetectors have been focused on the development of graphene hybrid structure such as graphene/semiconductors^10,11^, graphene/polymer^12^ or graphene/quantum dots^9,13^. In such structures, the photoelectric conversion usually occurs in the as-metioned introduced parts working as light harvesting layers or the junction generated between graphene and other semiconductors. The photo-generated charge carriers could shift into the graphene under the build-in field and change the conductance of graphene to achieve high responsivity. Among these hybrid structures, quantum dots stand out because of high-quality crystalline structures, solution processability, low cost, novel size-tunable wavelength and high responsivity^14,15^. Especially, colloidal semiconducting quantum dots (QDs) synthesized by chemical methods, which are tiny crystals of semiconductors with particle size as small as a few or several tens of nanometers, have attracted much attention for the light-emitting diodes and photosensitive devices^16,17^. For example, an infared photodetector based on CVD-grown graphene and PbS quantum dots has been proposed with ultrahigh responsivity of 10^7^ AW^−1^ at an incident power of about 30 pW^18^.

Recently, great progress has been made in the synthesis of QDs, and a large number of high quality QDs, such as CdSe, CdTe and CdS QDs, have been successfully synthesized by organometallic and aqueous approaches^19^. However, their efficient emissions are mostly limited in the range from the green to near-infrared spectral window due to a narrow band gap, which also limits the applications in short-wave band^20^. Another important factor, which hinders the further development of these QDs, is the toxicity of the Cd element. In contrast, the ZnSe QDs may be an excellent candidate for short-wave band appliactions due to their wide band gaps and low toxicity. Especially, when the surface of ZnSe is passivated by ZnS, a ZnSe/ZnS core shell quantum dot is formed and brings a remarkable enhancement of the optical properties compared with pristine ZnSe QDs^21,22^. The ZnS shell with higher energy can fill the detects on the surface of ZnSe QDs, resulting in a more perfect particle surface and an improved quantum yield. In addition, this core/shell structure also reduces the recombination of semiconductor nanocrystals and improves the stablity of QDs^23,24^. The synthesis methods for such ZnSe/ZnS core/shell quantum dots have been widely discussed and this class of materials has been applied as emitting materials in blue QD-LEDs due to their narrow emission peak and wavelength tunability^25–27^. However, their optoelectrical properties and potential applications in photodectors are rarely reported.

In this work, ultraviolet (UV) photoconductors based on CVD-grown monolayer graphene decorated with ZnSe/ZnS core/shell QDs by a simple solution method. The devices were fabricated on the bottom-gated Si substrates and exhibited a high reponsivity up to 2 × 10^3^ AW^−1^. The sensing mechanism is attributed to the photo-generated charge carriers in the QDs and the tranfer process from QDs to graphene, which may directly modulate the Fermi Level of graphene and the conductance of graphene channel. Besides, the ligand capping the surface of QDs, which is used to gurantee the stablity of QDs, was found to have an impact on the photo responsivity of QDs, especially the response/recovery time, due to its role in charger transfer process between graphene and QDs.

## Results

### Characterization of graphene/QDs FETs

**Figure 1:**
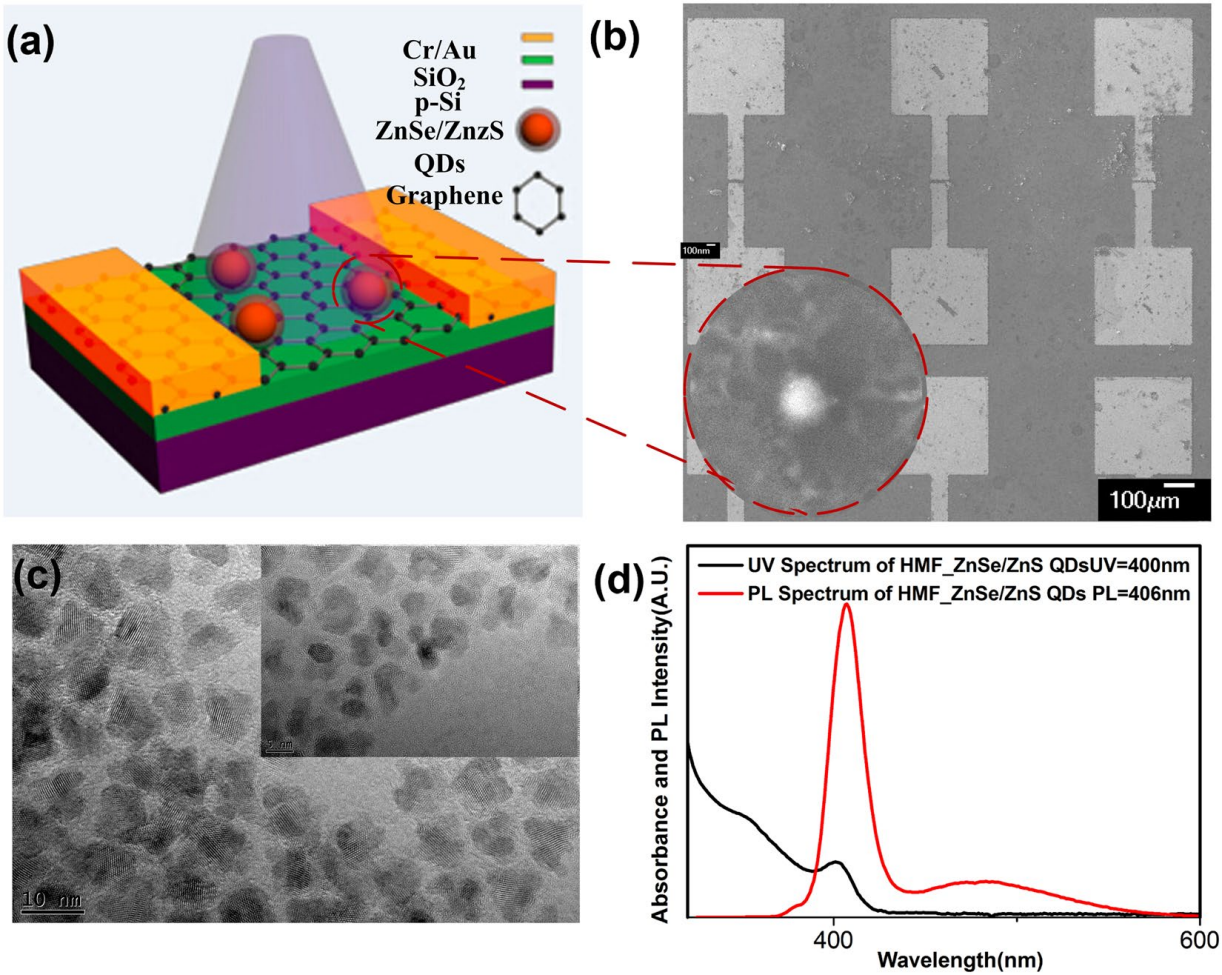
(**a**) Schematic illustration of a graphene FET decorated with ZnSe/ZnS quantum dots under the illumination. (**b**) The SEM image of our device arrays and the inset is the SEM image of ZnSe/ZnS quantum dots on the graphene films. (**c**) The TEM image of the ZnSe/ZnS quantum dots on the SiO_2_ substrate with different scale bars of 10 nm and 5 nm, respectively. (**d**) The UV spectrum of ZnSe/ZnS quantum dots with an absorption peak at 400 nm (black line) and the PL spectrum of ZnSe/ZnS quantum dots with an emission peak at 406 nm (red line).

The scanning electron micrograph (SEM) image of this device is shown in Fig. 1b, and the inset is the SEM image of a single ZnSe/ZnS QD in a spheroidal shape on the graphene channel, indicating a core-shell structure of ZnSe/ZnS QDs. Another SEM image of several QDs on the graphene channel with larger scanning rage reveals that the QDs were dispersed on the graphene (see Fig. S1 in supporting information). Meanwhile, an AFM image of these QDs on the SiO_2_ substrate also indicates a dispersed arrangement (see Fig. S2 in supporting information). In order to furtherly characterize the size of ZnSe/ZnS QDs, the transmission electron microscope (TEM) image of the QDs is shown in Fig. 1c with the scale bar of 10 nm and 5 nm, respectively. To examine the core-shell structure of ZnSe/ZnS QDs, the TEM images of pure ZnSe QDs and ZnSe/ZnS QDs are characterized, respectively (see Fig. S3a,b in the Supporting Information). The average sizes of QDs are measured to be from 3.5 to 4.8 nm for pure ZnSe QDs and from 5.3 to 6.5 nm for ZnSe/ZnS QDs, respectively, indicating a ZnS shell with thickness of ~1–2 nm. Moreover, the discrete spherical shape of ZnSe/ZnS QDs originating from the isolation effect of ZnS shell also gives the evidence on the core-shell structure of ZnSe/ZnS QDs. The optical properties of ZnSe/ZnS QDs have been investigated as shown in Fig. 1d. It can be found that these QDs exhibit an emission peak at 406 nm from the PL spectrum and an obvious absorption peak at 400 nm from the UV spectrum, which demonstrates the ZnSe/ZnS QDs may have a great potential application in optoelectronic devices, especially for UV ranges. Here, we mainly discuss the ZnSe/ZnS QDs/graphene hybrid structure as UV detectors, so an UV light with the wavelength of 405 nm will be adopted to examine the optical properties of our devices.

### Electrical and optical properties of graphene/QDs FETs

**Figure 2:**
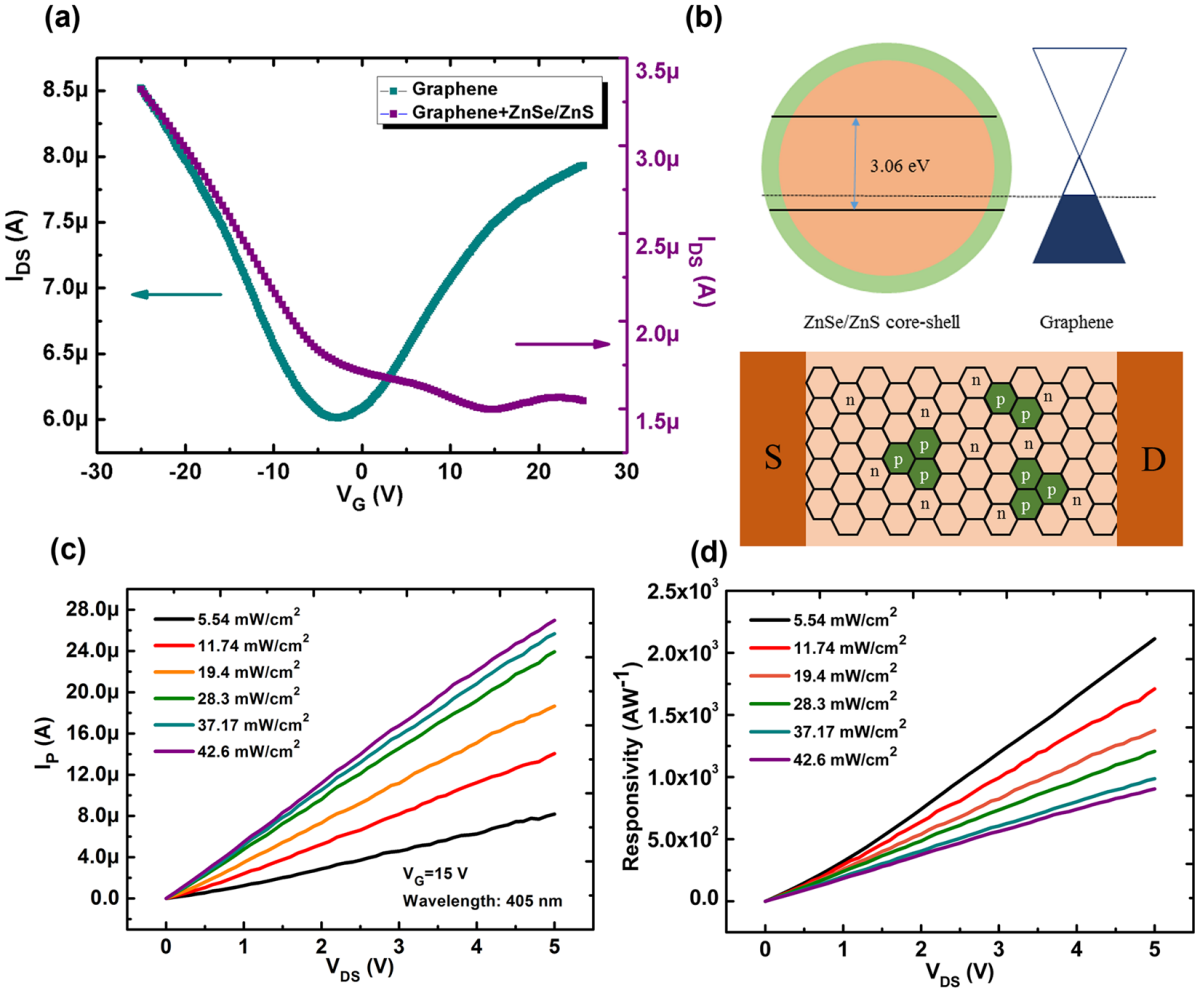
(**a**) The transport characteristics of graphene-based FET before (blue line) and after (purple line) the ZnSe/ZnS QDs spin-coated on the graphene surface. (**b**) Energy diagram of the heterojunction of graphene/ZnSe/ZnS QDs and graphene an doping distribution diagram of the ZnSe/ZnS QDs on graphene channel. (**c**) Photocurrent and (**d**) Responsivity of our devices as a function of the applied source-drain voltage (*V*_*DS*_) under the different light irradiation and wavelength of 405 nm.

The transport properties (*I*_*DS*_*- V*_*G*_ curves) of GFET was measured in the absence of light and is shown in Fig. 2a. Before the ZnSe/ZnS QDs spin-coated on the surface of graphene, the device shows a typical V-shaped transport curve, indicating that graphene exhibits an ambipolar field effect property. Then the ZnSe/ZnS QDs toluene solution is spin-coated on the graphene channl and the device is heated at 60 °C for 10 mins to evaporate the solvent for the further measurement. From Fig. 2a, the transport properties of the GFET modified by the QDs have exhibited W-shaped curve with several Dirac-like points. Besides, a obivous shift of Dirac point to a positive volgate indicates a p-type doping in graphene channel. In our previous works^28,29^, W-shaped curves in transfer characteristics of GFETs have been widely observed and the origin of such abnormal behaviors is due to the non-uniform doping in graphene channel induced by the adsorbate from ambient environment or the charge defects during the device fabrication process. Recent studies on II–VI group semiconductor nanostructures, such as ZnS and ZnSe NWs/NR, demonstrated that complementary doping, i.e., both n- and p-type doping, could be realized by carefully controlling the experimental conditions^30,31^. As shown in the SEM image (Fig. S1 in the supporting information), the QDs are scatterred across the graphene channel and the holes in p-type ZnSe/ZnS QDs will transfer to the graphene film and induce a local p-doping in graphene channel as shown in Fig. 2b. By this way, two kinds of junction structure may be built up between graphene/QDs and n-type graphene/p-type graphene, which needs larger *V*_*g*_ to get the charge neutral point and achieves at least two Dirac points. Then, the optoelectrical properties of our device was measured under the illumination with a wavelength of 405 nm. The photocurrent (*I*_*P*_) defined by *I*_*P*_ = *I*_*Light*_* − I*_*Dark*_, where *I*_*Light*_ is the drain current under the illumination and *I*_*Dark*_ is the drain current in the dark, and the responsivity to UV light was measured and calculated as a function of source-drain voltgate (*V*_*DS*_) in Fig. 2c,d, respectively. The photocurrent increases with the increase of *V*_*DS*_ and the light irradiation. The responsivity increases with the increase of *V*_*DS*_ but decreases with the increase of the light irradiation, which is consistent with the reported UV-detectors^32^. The maximum responsivity is about 2 × 10^3^ AW^−1^ at *V*_*DS*_ = 5V and an incident power of about 5.54 mW/cm^2^, which is a higher value compared with other previously reported graphene-based UV-detectors (Table 1). Moreover, the photocurrent response and corresponding responsivity under the different *V*_*G*_ was also investigated as shown in Fig. S4, demonstrating the gate-tunablity on the responsivity of such graphene/QDs.

**Table 1:** Performance comparison for UV detection between graphene/ZnSe/ZnS QDs-based phototransistors and the phototransistors based on other graphene hybrid structure.

Hybrid structure	Wavelength (nm)	R (A W^−1^)	Response time (rising)	Refs
G^a)^/ GaN nanowires	357	25	—	^40^
ZnO nanoparticle/Gcore–shell	375	640	9ms	^41^
ZnO QDs-doped G/h-BN/GaN	325	1915	6s	^42^
rGO^b)^/WO_3_ nanodiscs	347	6.4	0.35s	^43^
G/ZnO nanowires/G	365	23	3s	^44^
G/ZnSe/ZnS core/shell QDs	405	2000	0.52s	This work

^a)^G  =  Graphene, ^b)^rGO = reduced graphene oxide.

**Figure 3:**
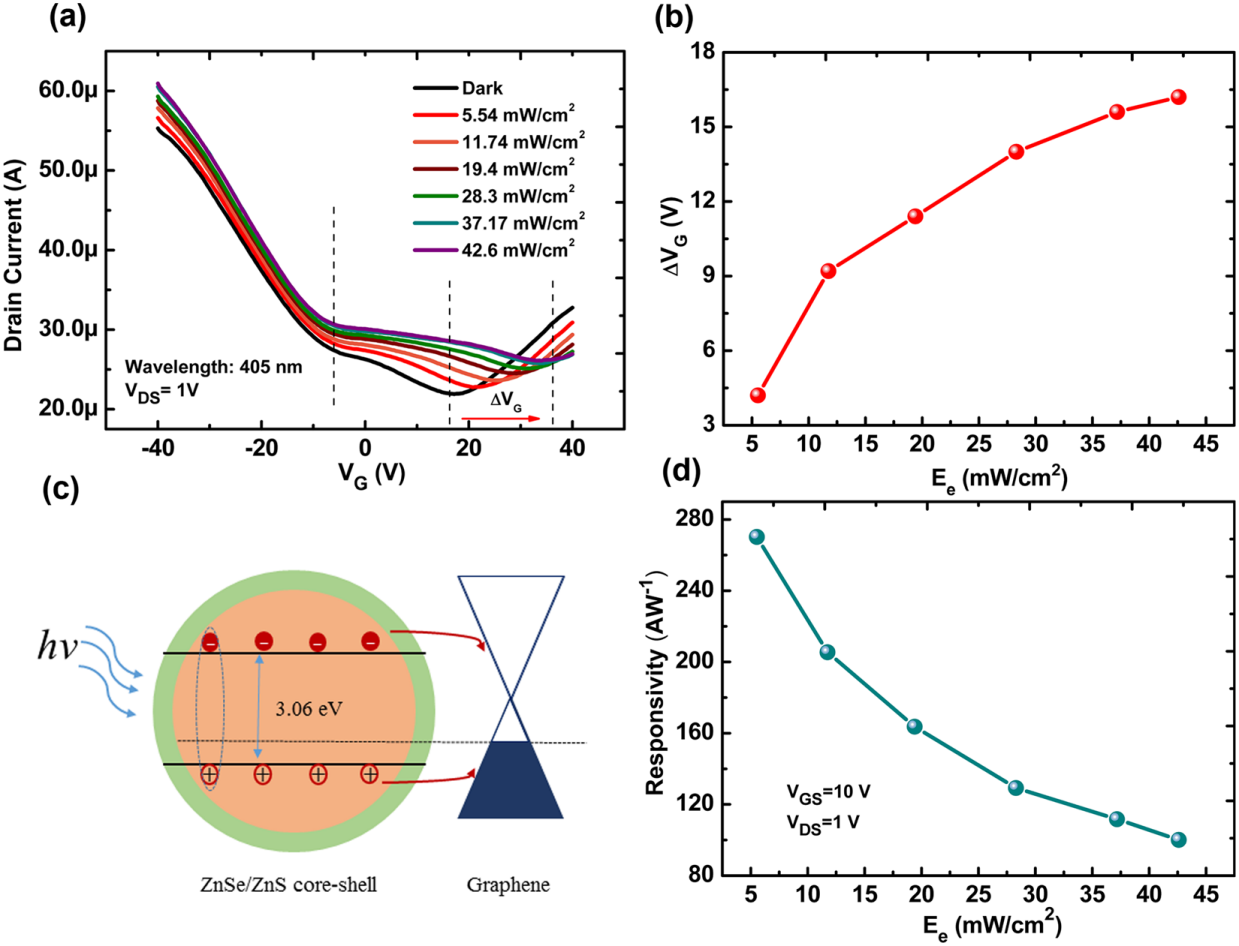
(**a**) Transfer characteristics of graphene-based phototransistors decorated with ZnSe/ZnS quantum dots under different light irradiation with the wavelength of 405 nm. (**b**) Horizontal shift of transfer curves as functions of light irradiation. (**c**) Schematic diagram for charge generation at a ZnSe/ZnS QDs/graphene heterojunction under light illumination. (**d**) Responsivity as a function of light irradiation with *V*_*GS*_ = 10 V and *V*_*DS*_ = 1 V.

To get insight into the sensing mechanism, the transfer characteristics of our phototransistor were characterized under light illumination at the wavelength of 405 nm with different irradiation. The transfer curves shift to higher positive gate voltage with the increase of light irradiation as shown in Fig. 3a. It is notable that with the increase of light irradiation (*E*_*e*_), the conductance of graphene channel increases for *V*_*G*_ < *V*_*Dirac*_, where the hole carriers are dominant, but decreases for *V*_*G*_ > *V*_*Dirac*_, where the electron carriers are dominant. In our work, ZnSe/ZnS QDs play an importance role in the photosensitive behaviors of our device, where a maximum photo responsivity is achieved at the wavelength of 405 nm compared with the results at the wavelength of 470 nm and 590 nm (see Fig. S5 in surporting information), which is consistent with results of absorption spectrum and PL spectrum in Fig. 1. In order to furtherly confirm the origin of such photo response, the devices based on pure graphene channel and pure ZnSe/ZnS QDs channel have been fabricated and their optoelectrcial properties have been investigated as shown in Fig. S6 (see the surporting information). Figure S6a shows there is little change of the current in dark and under the UV illumination for pure graphene-based FETs, indicating that the origin of photo response is the light absorption of QDs. While, the poor channel conductance of pure QDs-based FETs also demonstrates the important role of graphene as the photo-generated charge tranport channel (Fig. S6b). The shift of the transfer curves (*ΔV*_*G*_) is plotted as a function of light irradiation is shown in Fig. 3b, which demonstrates the photosensitivity of our GFETs. The sensing mechanisom for our photodetector could be attributed to the charge transfer process between ZnSe/ZnS QDs and graphene as shown in Fig. 3c. In graphene/ZnSe/ZnS QDs hybrid structure, ZnSe/ZnS QDs act as the light absorption layer and contribute to the conversion from photo to charge carriers. These photo-generated charge carriers would tranfer to the graphene due to the low energy levels for electrons and holes in graphene. However, the rate for the tranferred charges, electrons and holes, is different, which leads to a net holes or electrons in QDs. The origin of such difference for transfer process of electrons and holes may be due to the existence of surface chemical groups, for example the ligand used to seperate the QDs, which blocks the transmission of electrons to graphene channel^33,34^. From Fig. 3a, it can be inferred that under the illumination, the graphene is obviously p-doped, indicating an accumulation of holes in it. In this way, the number of holes tranferred to graphene is more than that of electrons and a net electrons in QDs will conversly induce more holes in graphene. A p-doped graphene may need a more positive gate voltage to achieve to the charge neutrality point and the accumulated holes in graphene would also result in a larger current through the channel. The similar process has been reported in a graphene/PbS QDs photodetector by Zhenhua Sun *et al*.^18^ Fig. 2d shows the responsivity of our device under the different light irradiation. It can be found that with the increase of light irradiation, the responsivity decreases in a nonlinear behaviors, which is similar to the most reported works^35,36^.

### Time-dependent characteristics of graphene/QDs FETs

**Figure 4:**
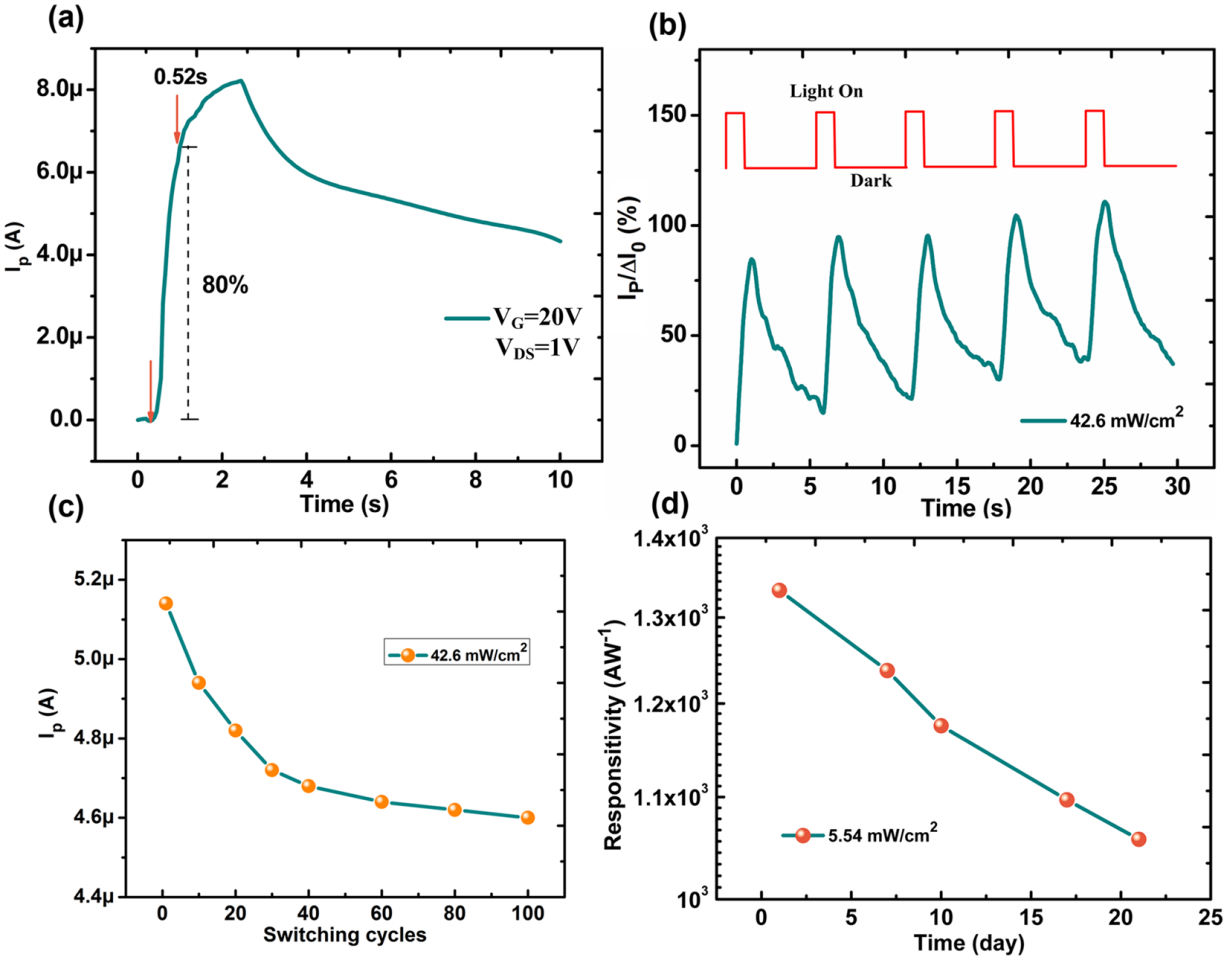
(**a**) Photocurrent response of our devices to periodic light switching on and off. *V*_*DS*_ = 1V, *V*_*G*_ = 20V, wavelength: 405 nm, light irradiation: 19.4 mW/cm^2^; (**b**) Normalized current response to on/off light illumination for several cycles. Light switch on time: 1s; Light switch off time: 5s. *ΔI*_*0*_ is the average maximum current response. (**c**) The photocurrent as a function of switching cycles. (**d**) The plot of responsivity vs placing time of our devices, which was placed in air as long as 21 days.

The transient behaviors with the illumination on and off and the reliability of graphene/QDs hybrid structure was investigated as shown in Fig. 4. The photocurrent increases with the illumination time and decreases with the time when the illumination is removed. The response time for the photocurrent increasing up to 80% of our device is found to 0.52s, which is relatively lower but comparable for other reported graphene-based photodetectors^18,37,38^. Acordding to the conversion mechanism from photo to charges talked above, the photo-generated charges need tranfer from QDs to graphene and collected to drain electrodes through the graphene channel. So, the response time includes the charges generation time, charges tranfer time between QDs/graphene and the charges collection time. It is notable that the charger generation occurs in QDs, which is a fast process due to a direct bandgap structure for ZnSe and the charge collection is achieved through the graphene channel, which offers a high carrier mobility. In this case, the slower response time may be due to the delay for the charge transfer from the QDs to graphene. The ligand capping the surface of QDs may be the origin because the oleic acid, ligand used here, is a long chain polymer, leading to an inefficient charger transmission. This issue may be solved by a ligand replacement technology by other short chain polymers, which may minimize the interparticle spacing to promote carrier transport^39^. Considering the complexity of ligand replacement technology, this improvement will be discussed in our future works. Figure 4b shows a repeated drain-source current response under the on/off illumination and the response of our device shows a similar trend after being switched one hundred times as shown in Fig. 4c. The stablity of our device is also characterized as shown in Fig. 4d and the responsivity is found to be more than 10^3^ AW^−1^ after being put in ambient for 21 days.

### Flexible graphene/QDs photoconductors

**Figure 5:**
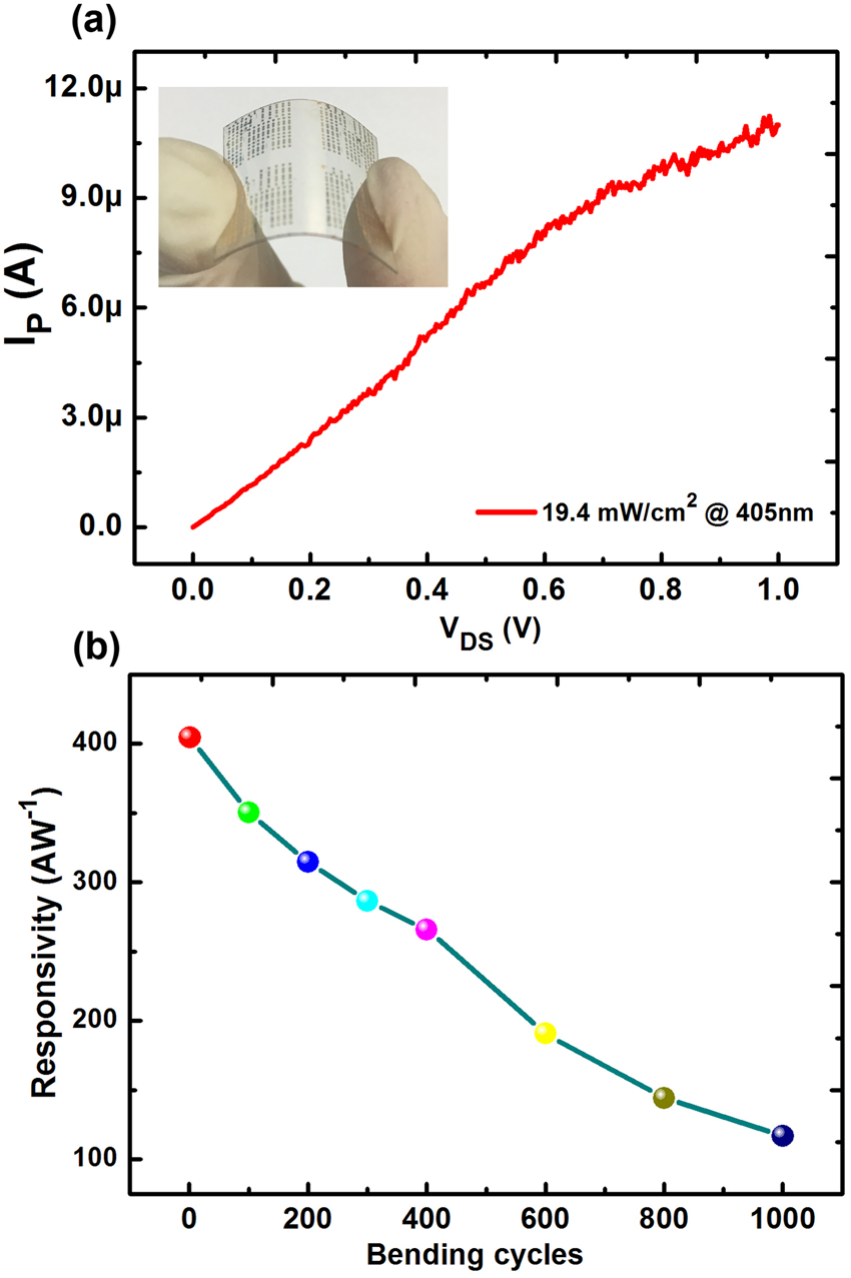
(**a**) The photocurrent of photoconductors based on ZnSe/ZnS QDs and graphene fabricated on the flexible PET substrate. Inset: a photograph of such flexible photoconductors; (**b**) The responsivity of such flexible photoconductors after a 1000 times bending test.

Considering the flexibility of graphene and QDs, such photoconductors could be fabricated on the flexible substrate as shown in the inset of Fig. 5a. CVD-grown graphene was transferred on the PET substrate followed by a standard lithography process to form the metal contact for photoconductors. Then, the ZnSe/ZnS solution was coated on the graphene-based photoconductor by a spin-coating method. Figure 5a shows the photocurrent of flexible graphene/QDs photoconductors under the illumination with the wavelength of 405 nm and the irradiation power of 19.4 mW/cm^2^. The photocurrent is promoted with the increase of irradiation, indicating a strengthed absorption of UV light when the bias voltage increases. The responsivity of flexible photoconductors was measured after a 1000 times bending test as shown in Fig. 5b. The results demonstrate that our flexible photoconductors remain to be functional with a responsivity about 100 AW^−1^ after the bending test. In this case, our device based on graphene/QDs can be easily fabricated on the flexible substrate and compatible with conventional CMOS technology to be pattern to smaller size, indicating a great potential in future wearable electronics.

## Conclusion

In conclusion, high responsivity UV photodetectors based on CVD-grown graphene decorated with ZnSe/ZnS core/shell quantum dots were realized by facile solution processing method. The ZnSe/ZnS QDs afford the conversion from photon to charges, which could be transferred from the QDs to graphene under the electric field of heterojunction between graphene and QDs. The responsivity of such photodetectors could be effectively tuned by the gate voltage and increases with the decrease of light irradiation. The maximum value reaches as high as 2 × 10^3^ AW^−1^ at a light irradiation of 5.54 mW/cm^2^ and *V*_*G*_ of 15V. Such photodetectors are found to dynamically response to periodic light switching on/off as many as 100 cycles and the response time of our devices is calculated to be 0.52s, which is limited by the ligand covering the surface of QDs. The optimization of ligand for QDs will be a crucial issue to further improve the response time of photodetectors. The responsivity of such photodetectors remains to be above 10^3^ AW^−1^ after being placed in air for 21 days. Moreover, flexible graphene/QDs hybrid photoconductors are realized and show an excellent flexible stability after 1000 times bending test, which confirms great potential of such low dimensional materials in future wearable and stretchable electronics.

## Method

### Fabrication of graphene/QDs FETs

Figure 1a shows the schematic of field effect phototransistor with graphene channel decorated with ZnSe/ZnS core/shell QDs dissolved in toluene (10 mg/ml, purchased from Mesolight Inc.). The bottom-gated FET structure has been adopted here with a highly doped p-type silicon as bottom electrodes and a 90 nm-thick SiO_2_ gate dielectric by thermal oxidation process as bottom gate dielectrics. Monolayer graphene grown by CVD method was transferred to the SiO_2_ substrate by a PMMA-assist transfer process to form the conducting channel. Two-step standard photolithography process was used to pattern the graphene channel and the S/D electrodes deposited by Cr/Au (10 nm/50 nm) through electron beam evaporation process. The ZnSe/ZnS quantum dots solution diluted to be 1mg/ml was spin-coated on the graphene surface and dried on the hot plate. Flexible photoconductors were fabricated on the flexible PET substrate and fabrication process was similar to that on SiO_2_ substrate.

### Characterization

SEM images were carried out on a Sirion-200 field-emission scanning electron microscope (FEI). TEM images were collected on a Hitachi H-7650 electron microscope operated at 80 kV. Absorption spectra were recorded on a Lambda 35 UV-vis spectrometer (Perkin Elmer). PL spectra were performed on a FLSP920 fluorescence spectrometer (Edinburgh). The electrical performance of the phototransistors was studied by using a B1500A Semiconductor Device Analyzer (Agilent Technologies) and Summit 11000 AP probe station (CASCADE microtech) at room temperature. The monochromatic lights with different wavelengths were provided by CEL-LEDS35 LED illuminant (CEAULIGHT). In the bending test, the flexible samples were bended to a radius of 7mm and bended up for 1000 times.

## Electronic supplementary material

Supporting Information
