# Supplementary material for: Hybrid graphene/cadmium-free ZnSe/ZnS quantum dots phototransistors for UV detection
Source: Sci Rep. 2018 Mar 23;8:5107. doi: 10.1038/s41598-018-23507-y (PMC5865151; doi:10.1038/s41598-018-23507-y)
Supplement: Supplementary file 1 — Supporting Information [file 41598_2018_23507_MOESM1_ESM.pdf]

Supporting Information

Hybrid graphene/cadmium-free ZnSe/ZnS quantum dots phototransistors for UV detection

Yi-Lin Sun[1], Dan Xie[1,*], Meng-Xing Sun[1], Chang-Jiu Teng[1], Liu Qian[2], Ruo-Song Chen[3], Lan Xiang[3], Tian-Ling Ren[1]

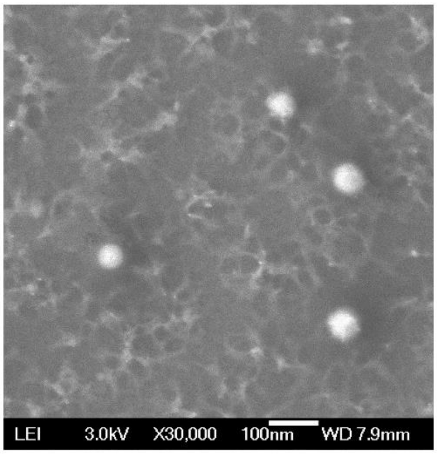

Figure S1. The SEM of ZnSe/ZnS QDs on the graphene channel.

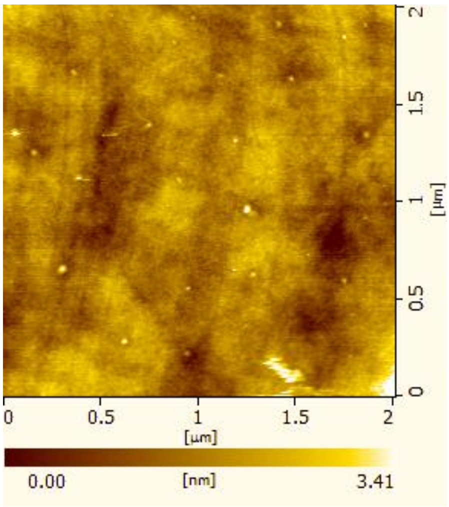

Figure S2. AFM image of ZnSe/ZnS quantum dots spin-coated on the SiO_2 substrate.

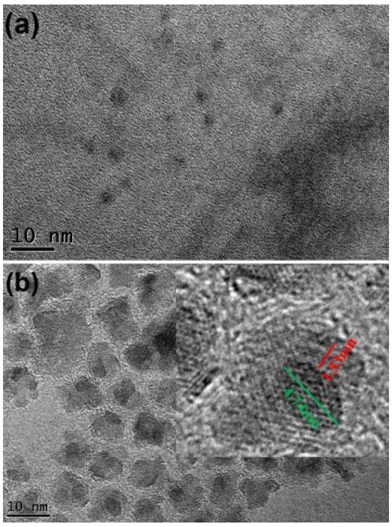

Figure S3. The TEM image of pure ZnSe QDs (a) and ZnSe/ZnS core-shell QDs (b). The inset of (b) is a single QD with core size of ~4.74 nm and shell size of ~1.53 nm.

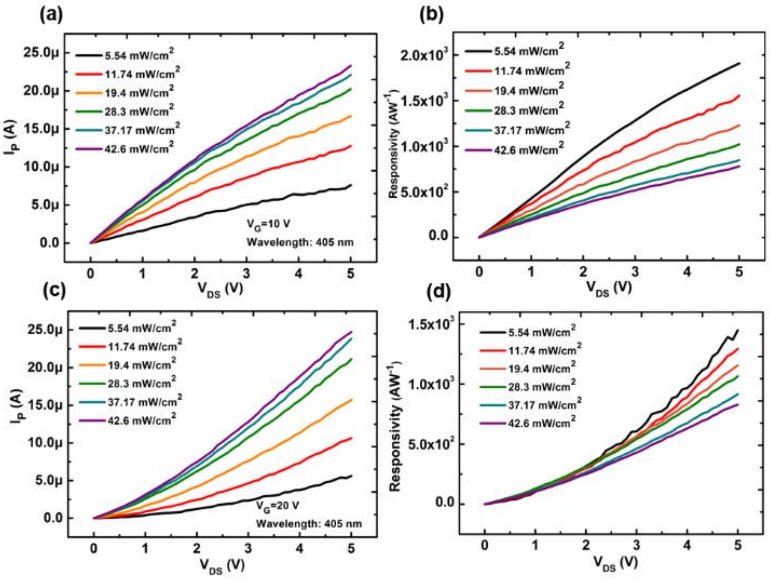

Figure S4. The photocurrent as a function of applied V_{DS} under the different light irradiations with V_G of 10 V (a) and 20 V (c); The responsivity of such graphene/QDs photodetectors calculated from (a) with a maximum value of 1964 AW^{-1} (b) and calculated from (c) with a maimum value of 1448 AW^{-1} (d).

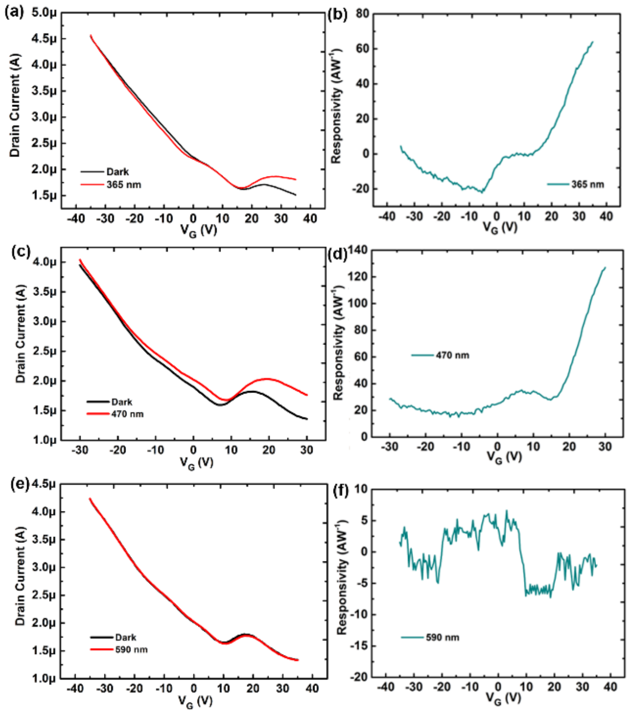

Figure S5. The transfer curves of graphene/QDs photodetectors decorated with ZnSe/ZnS quantum dots under the illumination with the wavelength of 365 nm (a), 470 nm (c) and 590 nm (e). The responsivity as a function of V_G with the maximum value of 64 AW^{-1} under the 365 nm light (b), 127 AW^{-1} under the 470 nm light (d) and 6 AW^{-1} under the 590 nm light (f).

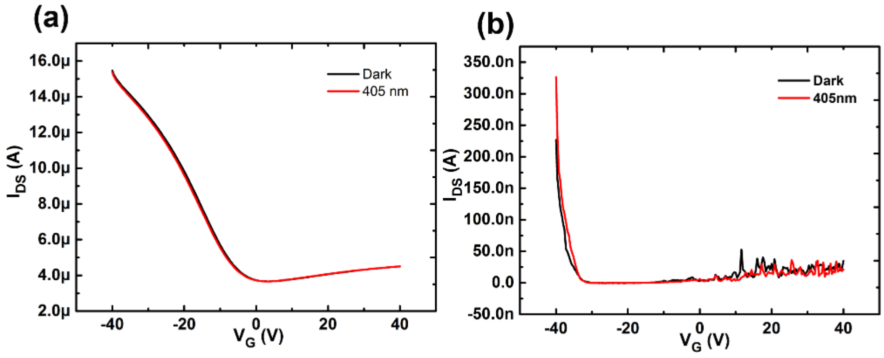

Figure S6. The transfer curves of pure graphene-based FETs (a) and pure QDs-based FETs (b) in the dark and under the illumination with the wavelength of 405 nm and irradiance of 11.74 mW/cm^2.
